# Epigenetic Regulation in *Hydra*: Conserved and Divergent Roles

**DOI:** 10.3389/fcell.2021.663208

**Published:** 2021-05-10

**Authors:** Anirudh Pillai, Akhila Gungi, Puli Chandramouli Reddy, Sanjeev Galande

**Affiliations:** Centre of Excellence in Epigenetics, Department of Biology, Indian Institute of Science Education and Research, Pune, India

**Keywords:** *Hydra*, Cnidaria, chromatin, RNAi, head organizer, enhancer, histone modifications, *cis*-regulatory elements

## Abstract

Transitions in gene regulatory processes responsible for the emergence of specialized cell types and spatiotemporal regulation of developmental signaling prior to the divergence of Cnidaria and Bilateria are poorly understood. As a sister group of Bilateria, the phylum Cnidaria can provide significant insights into these processes. Among the cnidarians, hydrae have been studied for >250 years to comprehend the mechanisms underlying their unique immortality and robust regenerative capacity. Studies on *Hydra* spp. and other pre-bilaterians alike have advanced our understanding of the evolutionary underpinnings governing eumetazoan tissue development, homeostasis, and regeneration. In addition to its regenerative potential, *Hydra* exhibits continuously active axial patterning due to its peculiar tissue dynamics. These distinctive physiological processes necessitate large scale gene expression changes that are governed by the multitude of epigenetic mechanisms operating in cells. This review highlights the contemporary knowledge of epigenetic regulation in *Hydra* with contemporary studies from other members of Cnidaria, as well as the interplay between regulatory mechanisms wherever demonstrated. The studies covered in the scope of this review reveal both ancestral and divergent roles played by conserved epigenetic mechanisms with emphasis on transcriptional regulation. Additionally, single-cell transcriptomics data was mined to predict the physiological relevance of putative gene regulatory components, which is in agreement with published findings and yielded insights into the possible functions of the gene regulatory mechanisms that are yet to be deciphered in *Hydra*, such as DNA methylation. Finally, we delineate potentially rewarding epigenetics research avenues that can further leverage the unique biology of *Hydra*.

## Introduction

### Epigenetic Regulation

Cells are the fundamental unit of life. Cell fate and function are determined by the biomolecular complement at homeostasis, which is responsive to the internal and external stimuli. The microenvironment experienced by a cell provides the cues necessary to determine its role relevant to the niche that it occupies. While the underlying genetic blueprint of life can be altered by mutations, the plasticity of its interpretation promotes the cellular heterogeneity characteristic of multicellular organisms. Once established, cellular physiology is stably inherited for cell type maintenance. Differentiated cells do possess lineage flexibility, evidenced by their direct (transdifferentiation) and indirect re-programmability (via a pluripotent state) to other cell types (Davis et al., 1987; Takahashi and Yamanaka, 2006). Epigenetic regulation, termed so for its overlying role in genome regulation, is fundamental to cell fate commitment (Waddington, 2012). Controlled and empirical studies have demonstrated that organisms can adapt to environmental stimuli like the availability of nutrition and stressors and that these adaptations persist over multiple generations even under attenuated selection pressure, which can cause altered physiology (Blewitt et al., 2006; Lumey et al., 2007; Carone et al., 2010; Chamorro-Garcia et al., 2013; Rehan et al., 2013; Öst et al., 2014; Carvan et al., 2017). Transgenerational epigenetic traits may be imprinted upon the offspring by either parent (Barton et al., 1984).

Epigenetics refers to the alterations in the flow of genetic information other than genetic ones that can be inherited over generations as defined by Waddington (2012). In this review, we use the term “epigenetic regulation” in a broader sense to describe changes that regulate the levels of gene expression at the level of both chromatin and RNA, without involving changes in the DNA sequence. Epigenetic regulation is affected at the molecular level by mechanisms that regulate the flow of genetic information–from genome to physiology–at various levels of transcriptional and post-transcriptional regulation. Regulators of transcription control the accessibility of DNA templates to RNA polymerases (Weintraub and Groudine, 1976), while post-transcriptional regulation impinges on RNA stability and translation. Genomic DNA in eukaryotes is condensed into chromatin via formation of an orderly complex with basic proteins called histones, for packaging within the dimensions of the nucleus as chromosomes (Wolffe and Kurumizaka, 1998). The structural unit of chromatin is the nucleosome, which comprises 146 bp of DNA wound 1.65 times around an octameric core of histones (Luger et al., 1997). Histone octamers generally consist of two subunits of each of the canonical core histones H2A, H2B, H3, and H4 (Kornberg, 1974; Ruiz-Carrillo and Jorcano, 1978) which, with the H1 and H5 linker histones stabilize nucleosomes to form higher-order chromatin (Allan et al., 1981). Variant versions of the core histones with specialized functions also exist that can get incorporated into nucleosomes (Talbert and Henikoff, 2021).

#### Histones and Histone Variants

Histones form the core for DNA compaction in the eukaryotic and Archaeal phyla and contribute in multiple ways toward regulating the activity of the transcriptional machinery. Across the animal kingdom, the level of conservation in the primary sequence and structure of histones is very high indicating their importance and a conserved mechanism of transcription, thereby offering multiple avenues to understand this process using various model organisms (Marino-Ramirez et al., 2011). The histone proteins H2A, H2B, H3, and H4 form a part of the nucleosome with DNA wrapped around it. This creates a nucleoprotein complex called chromatin in the cells for compaction and packaging of the genomic DNA. The core is formed by a complex of two H3–H4 heterodimers with a pair of adjacent H2A-H2B heterodimers (Eickbush and Moudrianakis, 1978). A fifth linker histone H1 binds to the sites of entry and exit of the DNA and is important for regulating the nucleosome repeat length, maintaining the higher-order structure of chromatin, and regulating gene expression (Fan et al., 2005; Woodcock et al., 2006; Hergeth and Schneider, 2015). The canonical histones all have few conserved features like occurrence in high copy numbers, a 3′ stem-loop structure, and high levels of expression in the S-phase of the cell cycle (Marzluff et al., 2008). In addition to the canonical isoforms the nucleosomal histones that compact the genome, there are multiple variants of the histones that play distinct roles in cells. The non-canonical variants have been evolutionarily classified as “universal” and “lineage-specific” based on their presence and function in different organisms (Talbert and Henikoff, 2010). The most common universal variants of H3 are CenH3 and H3.3 which have roles in assembling the kinetochore, transcriptional regulation, and germ-line specific chromatin remodeling (Ooi et al., 2006; Dalal et al., 2007; van der Heijden et al., 2007; Henikoff, 2008). The universal variants of H2A namely, H2A.Z and H2A.X play critical roles in transcriptional regulation and DNA damage response pathways (Zlatanova and Thakar, 2008; van Attikum and Gasser, 2009). A very important class of histone variants that evolved from the histone H1 is the protamines and protamine-like proteins. These are highly basic proteins which help in compaction and packaging of the chromatin in sperm cells (Eirin-Lopez and Ausio, 2009; Hammoud et al., 2009).

#### Transcriptional Epigenetic Regulation

**FIGURE 1:**
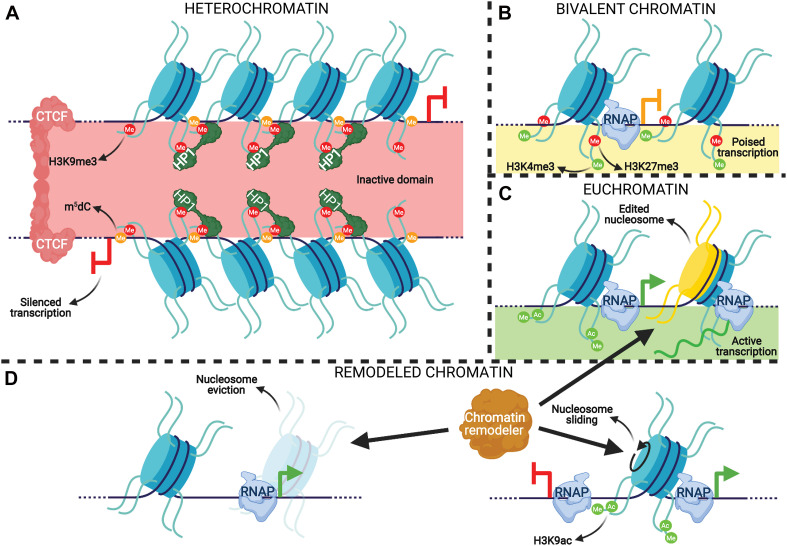
An integrative representation of chromatin-based transcription regulatory mechanisms. **(A)** A constitutive heterochromatin TAD demarcated by repressive modifications and CTCF. Nucleosomes (blue cylinders) associated with genomic DNA (dark blue string) are marked with the repressive H3K9me3 modification, which are recognized and bound by HP1α dimers. The DNA associated with heterochromatic nucleosomes is methylated at cytosines (orange circles on DNA), which reinforces gene silencing by H3K9me3 and vice versa to prevent transcription. The heterochromatin is “insulated” by CTCF dimers that prevent the repressive modifications from spreading to neighboring chromatin. **(B)** A promoter with bivalent nucleosomes. These nucleosomes are marked by both activating (H3K4me3) and repressive (H3K27me3) modifications that combinatorically poise RNA polymerase (RNAP) elongation. **(C)** Active transcription in euchromatin. Pause release of RNAP at the TSS of a gene is promoted by the activating modifications H3K4me3 and H3K9ac on the –1 nucleosome, promoting mRNA (green strand) generation. The +1 nucleosome of the gene is depicted as having histone variants incorporated (yellow half-cylinder), which is common for this nucleosome. **(D)** Functions of ATP-dependent chromatin remodelers. Remodelers can eject nucleosomes from DNA, slide them, or incorporate histone variants-such as at the +1 nucleosome in panel **(C)**.

The modification of nucleosomes is the basis of chromatin remodeling-a process that underlies transcriptional gene regulation. The various covalent modifications are acetylation, methylation, SUMOylation, citrullination, ubiquitination, ribosylation, phosphorylation, biotinylation, GlcNacylation, crotonylation (Tan et al., 2011), dopaminylation (Lepack et al., 2020), and serotonylation (Farrelly et al., 2019). In addition, they also undergo physical modifications like histone tail clipping (Duncan et al., 2008; Santos-Rosa et al., 2009) and proline isomerization (Nelson et al., 2006). Both covalent and physical modifications help in regulating transcription in cells. Although many types of histone modifications have been associated with transcriptional regulation, the well-studied modifications include the methylation and acetylation of specific lysine residues predominantly on H3 and H4 with many known roles in transcriptional regulation and cell fate determination. These modifications are respectively catalyzed or “written” by the lysine methyltransferase (KMT) and lysine acetyltransferase (KAT) category enzymes, and “erased” by the lysine demethylase (KDM) and histone deacetylase (HDAC) family members. While acetylation occurs singly on the histone residues, up to three methyl marks can be deposited on the lysine residues, and each level of methylation aids a specialized function. These individual modifications, their location on the chromatin and combinations thereof, collectively referred to as the “histone code” (Jenuwein and Allis, 2001), are known to enhance, repress and poise transcription. Acetylation of lysines on histones is associated with gene activation both by altering the chromatin physically and recruiting bromodomain-containing proteins and amplifying the activation signal across the length of DNA (Wade et al., 1997). In contrast to acetylation, methylation of lysines on histones does not have a universally activating or repressive function and has a greater level of specificity both in the role of the modification and the proteins that regulate the modifications. In the context of transcriptional activation, few histone marks have been established as markers of active transcription. The histone marks and the modifiers have been known to generate a cascade of events at gene promoters finally culminating in successful transcription of genes (Dou et al., 2005). H3K4me3 and H3K27ac have been classically associated with active transcription. While H3K4me3 occurs at the active promoters and promoter-associated non-methylated cytosine-phosphate-guanosine (CpG) islands of the genome (Hughes et al., 2020), H3K27ac is enriched at the active gene promoters and enhancers (Tafessu and Banaszynski, 2020). The level of enrichment on chromatin also helps in identifying regulatory elements on DNA (Heintzman and Ren, 2009). In addition to H3K27, acetylation of H3K9 also co-occurs with H3K4me3 at the promoters of actively transcribing genes. Although each type of histone modification has been ascribed a specific role, it is the combinatorial occurrence of the marks that determines the status of transcription in cells (Wang et al., 2008). Bivalent nucleosomes have both activating (H3K4me3) and repressive (H3K27me3) histone marks and transcriptionally poised chromatin (Bernstein et al., 2006). The various histone modifications are recognized by “reader” proteins that bind to a specific covalent modification and affect its function in gene regulation (Bartke et al., 2010; Vermeulen et al., 2010). Reader proteins were initially identified as bromo- and chromo-domain containing proteins which recognize acetyl- and methyl-marks on chromatin respectively (Dhalluin et al., 1999; Bannister et al., 2001). Later, with high-throughput screens, the Tudor and the MBT domain were identified as readers of the methylated lysine residue (Kim et al., 2006). The knowledge on domains that recognize the methylated lysines is continually increasing with PHD, chromo, WD40, Tudor, double/tandem Tudor, MBT, Ankyrin Repeats, zf-CW, and PWWP domains now being considered bona-fide readers (Yun et al., 2011). The Bromo domain is present in readers of the acetylated lysine residues and facilitates the recognition of multiple modified residues at one time allowing for large scale chromatin remodeling (Dhalluin et al., 1999). The various reader domains of the methyl mark obtain different levels of specificity by virtue of their binding pockets, the flanking amino acid residues around the target modified residue and their location on the histone peptide (Yun et al., 2011). The reading of the histone marks is a highly context dependent process and is necessary for chromatin modification, chromatin remodeling, maintaining chromatin architecture and recruiting other machinery important for many nuclear regulatory events. Physical alterations to chromatin are brought about by the ATP-dependent chromatin remodeler family of proteins that can either remove, reposition or incorporate histone variants into nucleosomes (Hyun et al., 2017). The higher-order structure and function of chromatin are controlled by the chromatin organizer proteins such as CCCTC-binding Factor (CTCF), Special AT-rich sequence Binding family proteins (SATB1/2), Yin Yang 1 (YY1), Krüppel-like Factors (KLFs), the pRb (retinoblastoma) protein (Longworth and Dyson, 2010) and cohesin (Rao et al., 2017). These well-characterized chromatin organizers bind to specialized DNA elements and guide the formation of chromatin loops (Galande et al., 2007; Ong and Corces, 2014; Weintraub et al., 2017; Di Giammartino et al., 2019). Chromatin organizer proteins interact with other transcriptional regulators including histone modifiers and chromatin remodelers to change the 3D landscape inside the nucleus. DNA also exhibits covalent modifications on cytosine and adenosine, particularly methylation at positions C-5 and N-6, termed m^5^dC and m^6^dA, respectively. m^5^dC is a context dependent regulator of transcription and its more commonly studied function is as a transcriptional repressor that commonly occurs at the CpG clusters of promoters of inactive genes and transposons, within the bodies of active genes to suppress spurious internal transcription, and also at insulator elements to block CTCF binding (Pennings et al., 2005; Tirado-Magallanes et al., 2017). There are recent reports suggesting the role of DNA methylation in enhancing transcription (Harris et al., 2018). It is catalyzed and mitotically maintained by DNA methyltransferases (DNMTs) while Ten Eleven Translocation (TET) proteins oxidatively remove the methyl moiety, via an hm^5^dC intermediate (Greenberg and Bourc’his, 2019). m^5^dC is recognized by Methyl-C_*p*_G-Binding Domain protein 2 (MBD2), which then recruits H3K9-specific KMTs and HDACs to repress transcription. Conversely, H3K9me3-bound Hetrechromatin Protein 1 (HP1α) can promote *de novo* DNA methylation by recruiting DNMT3A/B (Fuks et al., 2003a, b). In addition to the cytosine methylation on CpG islands, m^5^dC also occurs on non-CpG locations on the chromatin. Non-CpG methylation was first identified in plants and later in a few mammalian cell types (Becker et al., 2011). The studies on non-CpG methylation in other animal species are rare (Lucarelli et al., 2019). It has been found in ancestral species like *Chlamydomonas* (Feng et al., 2010), flatworm *Schistosoma mansoni* (Musto et al., 1994), and honey bees (Cingolani et al., 2013). It has specific functions in brain development and is enriched in many stem cell types and neurons and glial cells, although it is rare in most differentiated cell types (Patil et al., 2014; Jang et al., 2017). It is postulated that in the context of brain/neurons, non-CpG methylation could be vertebrate-specific (de Mendoza et al., 2021). In this manner, epigenetic marks can reinforce one another toward a convergent effect on gene expression via reader proteins. Unlike m^5^dC, m^6^dA is much lower in abundance in mammalian genomes and was found to silence young transposons on the X chromosome, as evidenced by their increased expression in m^6^dA demethylase (*alkbh1*) knockout mice (Wu et al., 2016). m^6^dA is positively correlated with transcriptional activation and transposon expression in *Drosophila*, *Caenorhabditis elegans*, *Chlamydomonas*, and fungi (Fu et al., 2015; Greer et al., 2015; Zhang et al., 2015; Mondo et al., 2017). In mitochondrial DNA, the mark is enriched and prevents transcription factor binding (Hao et al., 2020). ALKBH4 was also found to be an m^6^dA demethylase, while N6AMT1 and METTL4 were independently discovered to methylate deoxyadenosines (Xiao et al., 2018; Kweon et al., 2019). m^6^dA was also found to be passively incorporated during DNA replication in mammalian cell lines (Musheev et al., 2020). Adenosine methylation plays a suppressive role in mammalian gene regulation via Polycomb proteins, which are recruited by m^6^dA readers MPND and ASXL1 (Kweon et al., 2019). m^6^dA has been shown to activate transcription in some invertebrates (Luo et al., 2015). A graphical summary of the transcriptional gene regulatory mechanisms discussed here is rendered in Figure 1.

#### Post-transcriptional Epigenetic Regulation

**FIGURE 2:**
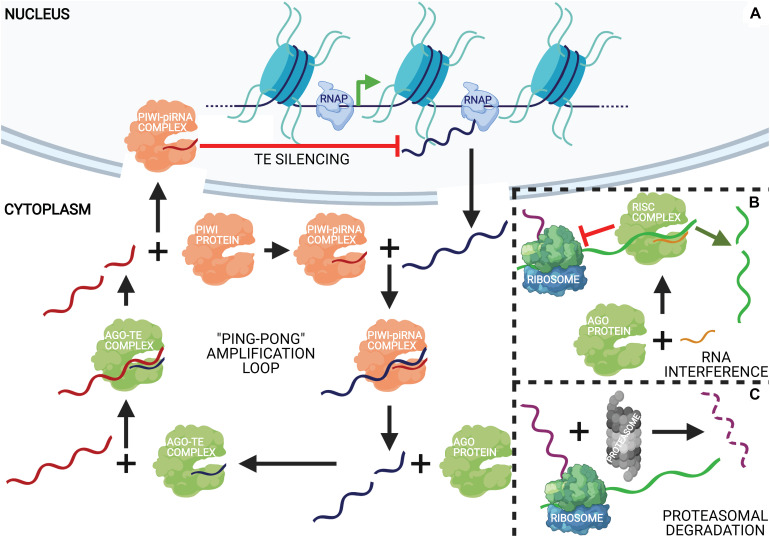
An integrative representation of post-transcriptional regulatory mechanisms. **(A)** Ping-pong piRNA biosynthesis and action. Argonaute (Ago) proteins incorporate fragments of transposable elements (TEs) (dark blue string) created by the PIWI-piRNA complex that cleaves them. The Ago-transposon complex cleaves piRNA precursors (red string) to produce mature piRNAs that get incorporated into PIWI proteins to complete the cycle, amplifying the production of mature piRNAs. The PIWI-piRNA complex can then translocate to the nucleus where it binds to transcribing retrotransposons complementary to the incorporated piRNA and represses them. **(B)** miRNAs (orange string) effect gene silencing through complexation with Ago proteins, forming RNA-induced silencing complexes (RISCs). RISCs can repress the translation of a complementary mRNA (green string) either by stalling ribosome elongation (left) or direct transcript cleavage (right), depending on the degree of miRNA-mRNA complementarity. **(C)** Protein downregulation is also an effect of degradation of the translated polypeptide (purple string) by the proteasome.

Pathways affecting the stability and translation of transcripts comprise the post-transcriptional tier of epigenetic regulation. This bottleneck on translation is caused by non-coding RNAs that range from 21 to 29 nucleotide (nt) in length, called small RNAs, which are generated by cleavage of a transcribed precursor RNA and are partially or fully complementary to their target RNA sequences. miRNAs and siRNAs are 21–23 nt long sRNA species that are recognized by and incorporated into RNA-induced silencing complexes (RISCs) that can bind to complementary mRNAs and either block their translation or inactivate them by cleavage (Gebert and MacRae, 2019). piRNAs are 22–35 nt long sRNAs that are typically complementary to transposons and repress them through P-element Induced Wimpy testis (PIWI) proteins, similar to RISC action (Kim et al., 2009). PIWI proteins can also transcriptionally silence transposons by binding to transcribing elements and recruiting DNMT3a for their *de novo* methylation, which is further enhanced and maintained with H3K9me3 (Aravin and Bourc’his, 2008; Aravin et al., 2008; Zoch et al., 2020; Wang and Lin, 2021). piRNA precursors, as well as those of mi/siRNAs, are transcribed from either independent genes or the loci of other genes/transposons (Gebert and MacRae, 2019; Ozata et al., 2019). A graphical summary of the post-transcriptional gene regulatory mechanisms discussed here is rendered in Figure 2.

### *Hydra* as a Model System

**FIGURE 3:**
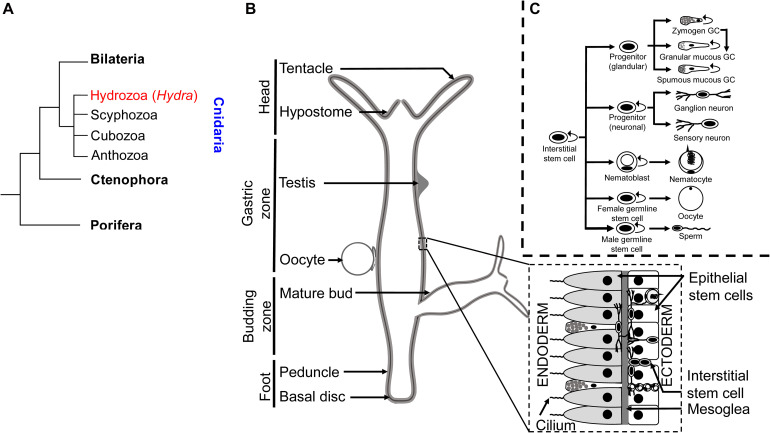
Taxonomy and anatomy of *Hydra* spp. **(A)** A phylogenetic tree showing the position of the phylum Cnidaria and the classes comprising. The genus *Hydra* lies within the class Hydrozoa, making it an early divergent metazoan order. **(B)** A labeled diagram of the external anatomical features of the *Hydra*. Note that *Hydra* exhibits sexual dimorphism, and therefore the depiction of an oocyte and testis on the same polyp is purely representational. A longitudinal cross section (inset) shows the two germ layers of *Hydra* and the most common cell types comprising the same. **(C)** Fate map of multipotent interstitial *Hydra* stem cells [adapted from Siebert et al. (2019)]. Interstitial cells are capable of yielding both germline and somatic cell lineages. Note that zymogen gland cells (GCs) can directly transdifferentiate into granular mucous GCs.

The phylum Cnidaria comprises marine and freshwater organisms with a radially symmetric body organized into two germ layers with tissue-level organization (Figure 3A). The phylum derives its name from the characteristic stinging cells called cnidocytes or nematocytes that its members possess for capturing prey. The genus *Hydra* comprises freshwater polyp species with a broad geographical distribution. Polyps comprise a columnar gastric region ending in the oral and aboral poles that are respectively organized into head and foot structures. The head comprises an oral pore, called the hypostome, through which prey is ingested, surrounded by 5–6 tentacles that harbor nematocytes to immobilize and capture live prey. A basal disk with mucus-producing gland cells forms the foot with which polyps adhere to substrata. The gastric column is composed of epithelial and interstitial stem cells and also harbors a diffused nerve net (Burnett and Diehl, 1964). Polyps exhibit sexual dimorphism and reproduce by external fertilization as well as asexually by budding (Figure 3B). Individual hydrae are composed of ∼120,000 cells that are distinguishable into 15–20 unique cell types (Bode et al., 1973; David, 1973). Recent advances in sequencing technologies have facilitated further identification of 12 neuronal subtypes in *Hydra* and the molecular determinants of the trajectories of interstitial cell differentiation (Siebert et al., 2019; Figure 3C). Continuous shedding of differentiated cells occurs at the anteroposterior termini, with the gastric pool of stem cells actively dividing and differentiating to maintain tissue homeostasis (Campbell, 1967). Direct transdifferentiation of *Hydra* cells has also been demonstrated *in vivo*, in response to morphogen gradients originating from organizer cells in the head (Siebert et al., 2008). The head organizer function of the hypostome was first demonstrated by grafting the hypostome onto the gastric column, resulting in ectopic body axis formation (Browne, 1909). Hypostomal cells secrete the Wnt ligands as the head-promoting morphogen (Hobmayer et al., 2000), whereas NK2 is a basal disk-specific factor that promotes foot organization (Grens et al., 1996). Stem cell maintenance is promoted by the transcription factor FoxO (Boehm et al., 2012).

#### Regeneration in *Hydra*

Hydrae are renowned for their robust regenerative capacity, with the ability to reaggregate after dissociation into their constituent cells (Trembley, 1744; Gierer et al., 1972). Polyps regenerate by morphallaxis which relies on the transdifferentiation of cells to replace lost ones (Cummings and Bode, 1984). The relatively simple morphology of *Hydra* makes it an ideal system to study tissue development, homeostasis, and regeneration. However, gene knockout studies remain absent to date in *Hydra*, and hence RNAi is more commonly employed for gene silencing (Lohmann et al., 1999). Furthermore, cell culture using cells derived from *Hydra* has not yet been established. The emerging technique of single-cell RNA sequencing (scRNA-seq) has been utilized to understand the transcriptomes in heterogeneous populations of cells in tissues and animals. The single cell RNA sequencing along with transcript localization studies have provided significant insights into the cell type specific transcriptional programs, body plan patterning and also led to the identification of novel cell-types in cnidarians such as *Xenia*, *Nematostella*, and *Hydra* (Sebé-Pedrós et al., 2018; Hu et al., 2020). Siebert et al. recently performed scRNA-seq on *Hydra vulgaris* AEP by dissociating polyps and individually sequencing ∼25,000 cells, thereby generating an atlas of gene expression across the constituent cell types in their various molecular “states.” Analysis of the dataset confirmed previous results, such as the head organizer-specific expression of *wnt3a* and the graded downregulation of *dickkopf-like 1/2/4 C* during the transdifferentiation of zymogen gland cells to granular mucous gland cells (Siebert et al., 2019). We have mined this dataset for the expression levels of confirmed and predicted orthologs of epigenetic regulators in *Hydra*, and the cell types found to have the different levels of expression of each ortholog were noted. Only mRNAs were queried as sRNAs are unavailable in the dataset. Observing the mRNA levels of putative epigenetic modifiers across different cell states can indicate their physiological role, though the corresponding protein expression profile is more predictive.

Nearly all queried orthologs are found to be highly expressed in stem cells while low in terminally differentiated cells, suggestive of convergent roles in differentiation. In addition, many of the epigenetic regulators show very little expression in the ectodermal and endodermal cells of the interstitial lineage. The cell type specific expression is predictable using this database but there are a few exceptions wherein when the expression is below the threshold set by the analysis parameters it is not visible in any cell type. These genes could be expressed in a context specific manner when certain complex physiological processes such as regeneration are triggered. Studies specifically addressing these processes will provide more insight into the role of these genes. The use of state-of-the-art technologies and the ability to generate transgenics in organisms such as *Hydra* whose phylogenetic position as a sister group member of Bilateria, allows us to understand the ancestral roles of epigenetic regulators (Wittlieb et al., 2006; Klimovich et al., 2019; Figure 3A). Current knowledge on the regulation of gene expression at the transcriptional and post-transcriptional level in *Hydra* and other early diverging metazoans has been summarized in this review.

**FIGURE 4:**
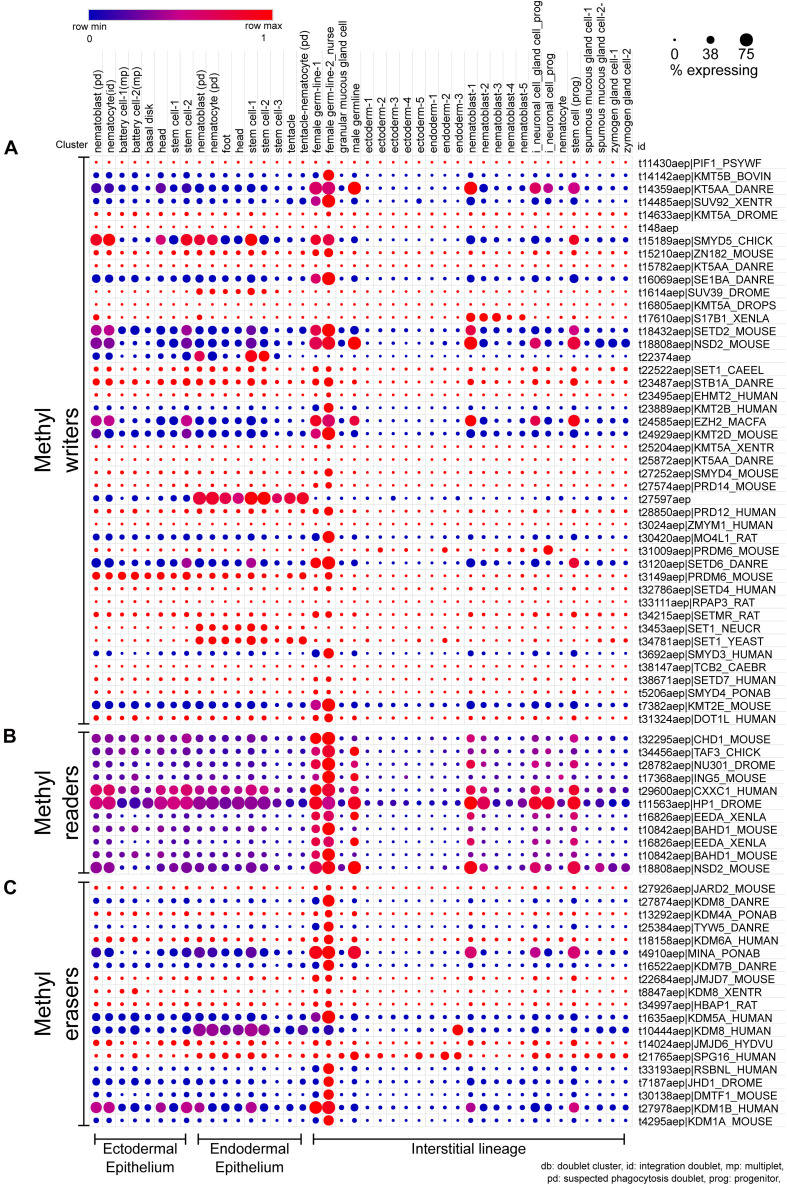
scRNA-seq dot plot depicting the expression profiles of various proteins that regulate the methylation of lysines on histones. **(A)** The writers include methyltransferases harboring either the DOT domain or the SET domain as the catalytic domain. **(B)** The chromodomain proteins that recognize the methylated lysines and recruit further interactors are the methyl readers. **(C)** The erasers include proteins harboring either the AOD domain or the JmjC domain acting as the catalytic domain. The dot plot visualized here depicts the cell types on the *x*-axis as labeled on the top. The dot color represents the expression level, and the legend is provided above the plot. The dot size represents the % of cells in each cluster expressing the respective gene. The cell types have been grouped based on their lineage in the polyps.

**FIGURE 5:**
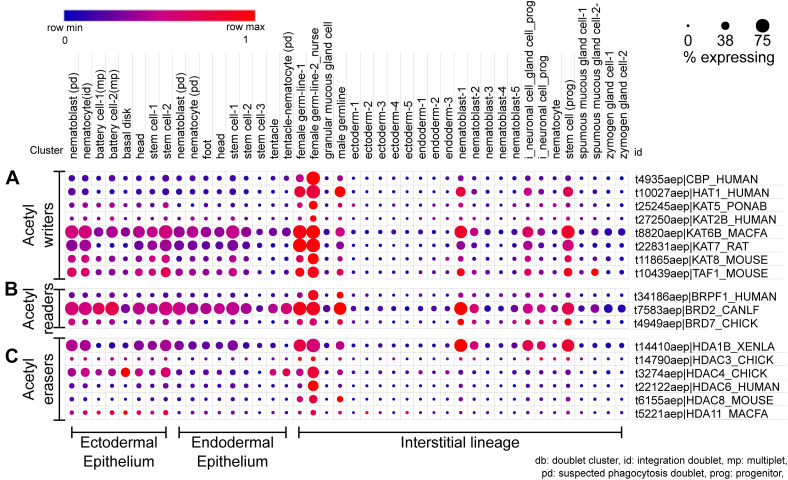
scRNA-seq dot plot depicting the expression profiles of various proteins that regulate the acetylation of lysines on histones. **(A)** The writers include acetyltransferases with the catalytic HAT domain. **(B)** The bromodomain proteins that recognize the acetyl mark and recruit further interactors are the acetyl readers. **(C)** The erasers include proteins harboring the HDAC domain. The dot plot visualized here depicts the cell types on the *x*-axis as labeled on the top; The dot color represents the expression level, and the legend is provided above the plot; The dot size represents the % of cells in each cluster expressing the respective gene. The cell types have been grouped based on their lineage in the polyps.

**FIGURE 6:**
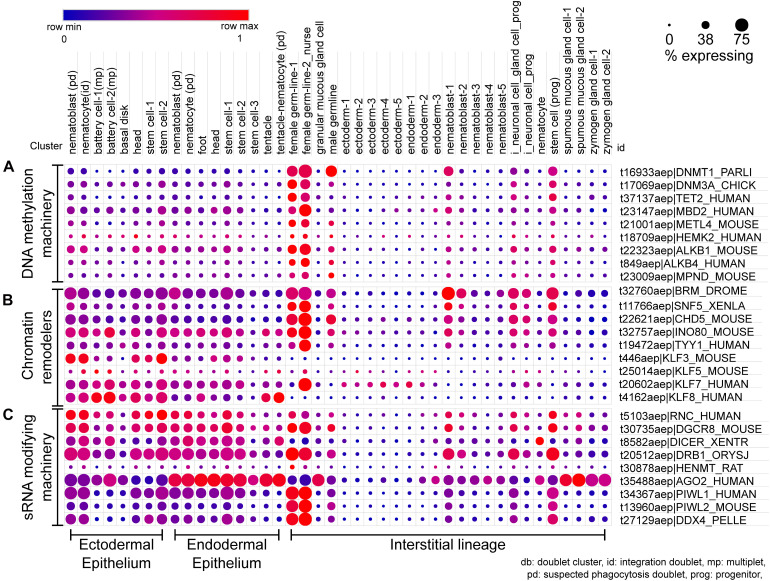
scRNA-seq dot plot for the expression profiles of various classes of epigenetic regulators. **(A)** The profile of DNA methylation machinery including the methyltransferases, demethylases, and DNA methyl reader proteins. **(B)** The dot plot represents the expression profile of the different chromatin remodelers which interact with readers, writers, and erasers and bring about large-scale changes in the chromatin. **(C)** The cell type-specific expression of small RNA modifying machinery for both RNA interference and piRNA processing is depicted in this dot plot. The dot plot visualized here depicts the cell types on the x-axis as labeled on the top; The dot color represents the expression level, and the legend is provided above the plot; The dot size represents the % of cells in each cluster expressing the respective gene. The cell types have been grouped based on their lineage in the polyps.

Among the different cell types, nurse cells exhibit the highest expression for ∼78% of the queried mRNAs (Figures 4–6), suggesting that they may either exhibit highly dynamic chromatin architecture and/or act as maternal transcript and protein stores due to the high levels of transcription, which is critical for their role in ooplasm contribution (Alexandrova et al., 2005). Additionally, many of the epigenetic regulators are also highly expressed in germ cells (Figures 4–6). In *Hydra* the multipotent I-cells give rise to primordial germ cells (PGC) which eventually give rise to male and female germ cells (Littlefield, 1985; Bosch and David, 1987). Sexual reproduction in *Hydra* is controlled by external environmental cues such as temperature and they also exhibit sex reversal (Littlefield et al., 1991; Nishimiya-Fujisawa and Kobayashi, 2012). This occurs in adult life, unlike in bilaterians where this process occurs in early embryonic development. This provides a unique opportunity to study the emergence of the sex determination process and associated molecular regulation. However, to date, the underlying molecular regulation involved in the sex determination process of *Hydra* is not determined completely. In mammals, a tripartite network of BLIMP1, AP2γ, and PRDM14 plays a critical role in PGC specification. Among these BLIMP1 and PRDM14 regulate various epigenetic regulators such as KMT (*Ehmt1*-homolog of KMT1D), KDMs (*Kdm43a*, *Kdm4b*, and *Kdm6b*), DNMTs (*Dnmt1*, *Dnmt3a*, and *Dnmt3b*), and HDACs (*Hdac4* and *Hdac7*) (Magnusdottir and Surani, 2014). These epigenetic modifiers play a role in reprogramming the somatic cells to PGCs where both “naïve” states and imprinted states are achieved and transmitted to the next generation upon reproduction (Hajkova, 2011; Hill et al., 2018). Observed highly expressed epigenetic regulators in *Hydra* germ cells might play a similar role in PGC determination and development. This requires a thorough characterization of the epigenetic modifiers found in *Hydra*.

The phylogenetic position of cnidarians together with the dynamic tissue turnover and regenerative capacity of *Hydra* make it a useful model system for tracing back the evolutionary trajectories of the underlying mechanisms of gene expression regulation. Experiments on polyps have yielded insights into how the conserved cellular coordination is affected in a minimalistic body organization, remains conserved in vertebrates, and impacts the unique physiology of *Hydra*. Studies elucidating the epigenetic mechanisms operating at the transcriptional and post-transcriptional level in *Hydra* polyps are discussed in this review.

## Chromatin Regulatory Components in *Hydra*

The genome of *Hydra* is highly AT-rich (71%) and comprises a large proportion of transposable elements (57%) (Chapman et al., 2010). The size of *H. vulgaris* genome is 1250 Mbp and is made up of 15 pairs of chromosomes with no variation (Zacharias et al., 2004). No obvious sex chromosomes have been identified in *Hydra* till date. The *Hydra* genome comprises ∼20,000 protein-coding genes and a minimum of 238 miRNAs (Chapman et al., 2010; Krishna et al., 2013). Additionally, transcriptome analysis resulted in the identification of approximately 81 probable long non-coding RNAs (lncRNAs) (Wenger and Galliot, 2013). The *Hydra* genome is compacted and the gene expression is regulated by nucleosomes as observed in other animal species.

### Histone Variants

There are only a few studies on histone variants in *Hydra* so far. The chromatin of interstitial cells in *Hydra cauliculata* was found to be enriched with canonical histones during somatic and male germline differentiation, concomitant with condensation. Spermatocyte formation is accompanied by a transition from lysine-rich to arginine-rich chromatin, similar to the replacement of nucleosomes by protamines in higher metazoans during spermatogenesis. In *Hydra hymnae*, which has arginine-rich histones, this transition does not occur. Moreover, protamine transitions were not detected in either species (Moore, 1971; West, 1978). Indeed, *Hydra* spp. do not encode protamines but a variant of H2B called H2B.6 that presumably plays role in DNA compaction during spermatogenesis has been identified similar to multiple H2B variants in *Hydractinia echinata* (Török et al., 2016; Reddy et al., 2017). The genomic organization of the histone repertoire encoded by *H. vulgaris* Ind-Pune has been mapped. Each canonical core histone is encoded by an average of ∼17 gene copies, contrasted with only one gene per variant and for histone H1, which is clustered with single copies of the four core histones in a quintet organization common to cnidarians. Among the variants identified for H2A were two H2A.X-type histones, which are known to play a role in the early DNA damage response. Both these variants, but not canonical H2A, were upregulated during bleomycin-induced DNA damage. This result, together with their higher expression in the gastric column, suggests a role for H2A.X in protecting the constantly dividing genomes of *Hydra* stem cells. Other histone variants identified include H2A.Z, macroH2A, H3.3, and CENP-A homologs. Phylogenetic analysis revealed high amino acid variation across cnidarian H2A and H2B histones, particularly between *H. vulgaris* and *H. echinata*, suggesting that they diverged within Hydrozoa (Reddy et al., 2017). The replication dependence of the histones was determined by either fluorescent labeling or the presence of a 3′-UTR stem-loop characteristic of replication-dependent histone mRNAs, whereas their expression patterns were deduced by *in situ* hybridization. Notably, a replication-dependent H3.3 variant was identified that is speculated to play a role in bookmarking transcriptionally active chromatin (Török et al., 2016). The replication dependency and functions of histone variants other than H2A.X are yet to be characterized in *Hydra*, but their presence in polyps suggests that they may play broadly conserved roles in genome regulation.

### Histone Modifiers and Modifications

Among the various histone modifications and modifiers, studies in *Hydra* have dealt with histone methylation and acetylation. Typically, these are well-established marks on histones that are associated with transcriptional regulation. In this review, we have focused on these marks and their modifiers including writers and erasers.

#### Histone Methylation

Early studies used *in situ* hybridization for localizations of epigenetic modifiers and immunofluorescence to identify their target modifications. The modification H3K27me2/3 and its writer EZH2 are enriched in interstitial cells and male germline cells (spermatogonia and spermatocytes) in *Hydra*. HyEED, encoding a homolog of the mammalian Embryonic Ectoderm Development (EED), which is a core component of the Polycomb Repressive Complex 2 (PRC2) complex and critical for the H3K27me2/3 mark was upregulated during embryogenesis in *H. vulgaris*. The modification in addition to the histone variants may serve to silence sperm chromatin in the absence of protamines, thereby playing a conserved role in a divergent trait. The H3K27me2/3-positive population may correspond to primed stem cells owing to an enriched presence in the interstitial cells over their derivative somatic cells (Genikhovich et al., 2006). H3K27me2/3 was strongly enriched in nematoblasts confirming the function of PRC2 in interstitial cell differentiation to nematocytes *in vivo*. Further, EED overexpression was countered by proteasomal degradation to retain the wild type EED expression pattern, suggestive of position-dependent proteostasis in the body column of *Hydra* (Khalturin et al., 2007). An investigation into the localization of *Hydra* PRC2 complex proteins EED and EZH2 revealed the encoding mRNAs to be highly expressed in the interstitial cells, whereas the corresponding proteins were enriched in the ectodermal epithelial cells. H3K27me3 *per se* was enriched in interstitial cells. This study also found that *Hydra* YY1 directly interacts with PRC2, raising the possibility that *Hydra* YY1 may play an evolutionarily conserved role in PRC2 recruitment for targeted gene repression. Interestingly, the YY1 protein was detected in nuclei of ectodermal epithelial cells, though their membranes also stained positive. It is therefore plausible that PRC2 recruitment plays a role toward the maintenance of this cell lineage as well (Matt, 2011). A conserved localization at open chromatin regions was observed for H3K4me2 and H3K4me3 in whole and head-regenerating polyps and higher levels of H3K4me2 were correlated with the enhancer regions of the *Hydra* genome. However, the typical higher enrichment ratios did not predict the promoter and a potential enhancer of the *wnt3a* locus (Murad et al., 2019). A recent study from the Galande laboratory using chromatin immunoprecipitation (ChIP)-sequencing found that the intergenic regions constitute the majority of H3K27ac- and H3K4me3-associated DNA followed by the +1 nucleosomes which positively correlate with the *cis*-regulatory elements and the transcription start sites (TSSs) respectively. The presence of a regulatory switch based on the acetylation/methylation on the H3K27 residue which is critical for Wnt/β-catenin mediated axis patterning in *Hydra* has been elucidated (Reddy et al., 2020b). H3K4me3 also positively correlates with RNA levels in *Nematostella vectensis* and co-localizes with the +1 nucleosomes, although it has not been detected at any of the putative enhancers. In contrast, H3K4me1/2 modifications are enriched at the enhancers, with H3K4me2 levels concordant with the RNA levels. Interestingly, detection of RNA Pol II at the *Nematostella* enhancers suggests enhancer-promoter looping and enhancer RNA transcription (Schwaiger et al., 2014; Duttke et al., 2019). Enhancer RNA transcription is also indicative of active enhancers and active promoter-enhancer interactions (Kim et al., 2015). It is plausible that the cnidarian stem cells possess the bivalent chromatin modification signature that keeps lineage-specific genes in a transcriptionally poised state, the confirmation of which would require the detection of H3K4me3 and H3K27me3 co-enrichment using techniques such as sequential ChIP. It may also be of interest to determine how the H3K27me3-negative interstitial cell population differs from those positive for the modification. The role of histone lysine demethylase LSD1 that targets H3K4me1/2 has also been elucidated in the development and differentiation of cnidarian-specific neural cells in *Nematostella* (Gahan et al., 2020). The expression pattern of the various components of the modification machinery involved in writing, reading, and erasing the methylation of histones in different cell types of *Hydra* is shown in Figure 4. In addition to the male germline cells which were previously shown to have EZH2 expression, scRNAseq data analysis also led to the identification of female germline stem cells and the different types of interstitial stem cells or progenitor cells to have expression of this gene. EED which was upregulated during embryogenesis was detected in the female and male germline stem cells and to a lesser extent in few interstitial progenitor cell types. Overall, the scRNA-seq data corroborates the expression data on EZH2 and EED from the previous studies (Figure 4A).

#### Histone Acetylation

Histone H3 lysine 27 acetylation (H3K27ac) was found to be a more reliable signature of enhancers in *Hydra* and was also enriched at the open promoter-proximal regions, consistent with its role in transcriptional activation. To understand the dynamics of H2K27ac occupancy during regeneration, ChIP-sequencing was performed on chromatin isolated from regenerating tips of *Hydra* at various time points. H3K27ac was most enriched at the putative *wnt3a* enhancer in the head region and the promoter-proximal region 4 h post-decapitation (Murad et al., 2019) and exhibits a conserved occupancy and transcriptional correlation like H3K4me3. H3K27ac increases at the putative enhancers of head-related genes upon disruption of axis patterning, suggesting that H3K27ac activates axis patterning enhancers that are repressed by H3K27me3 in other tissues (Reddy et al., 2020a). A similar occupancy-to-expression trend is observed for H3K27ac in *N. vectensis*, which also correlated with the occupancy of the acetyltransferase p300 (Schwaiger et al., 2014). Histone hyperacetylation by HDAC inhibition results in diminished head and foot boundaries, attributed to the de-repression of the cell cycle genes in these differentiated regions. Bud detachment failure is also observed, consistent with a foot boundary defect, which eventually promotes ectopic body axes. HDAC activity *in vivo* is predicted to be dependent on phosphatidylinositol, based on the marked enrichment of its biosynthetic pathway components in the head and foot and HDAC enrichment in the body column. Therefore, histone acetylation plays an important role in stem cell maintenance (López-Quintero et al., 2020). In a closely related hydrozoan, *Hydractinia*, a homolog of HDAC1/2 (*Hydractinia* Hdac2) has been shown to play a key role in the regulation of neurogenesis and also in regeneration. Here, it has been demonstrated that Hdac2 interacts with SoxB2 and regulates the differentiation of neurons from stem cells (Flici et al., 2017). Later it has been reported that the inhibition of Hdac2 affects the migration of proliferative cells and the formation of a blastema, thus affecting regeneration (Flici and Frank, 2018).

The scRNA-seq data revealed that the canonical HAT CBP is mainly expressed in the female germline cells (Figure 5A). Other acetyltransferases appear to be expressed in stem cells and differentiated cell types of all lineages and could be playing a role in taxon-specific functions in *Hydra*. There have been no studies that investigate the expression patterns of the reader and eraser proteins of histone acetylation and few predictions can be drawn from the scRNA-seq data for future studies. The BRD2 protein shows an expression in multiple cell types with higher levels in progenitor cell types which could be a predictor of its function. Similarly, among the various HDAC proteins present in *Hydra*, the HDAC1 homolog shows expression in various differentiated and stem cell types of all three lineages (Figure 5C). It will be useful to understand how active enhancers in the head retain acetylation in the background of HDAC activation.

## DNA Methylation in *Hydra*

DNA methylation in *Hydra* is yet to be functionally characterized, with only its genomic and transcriptomic presence quantitatively determined so far. The total nucleotide composition of the genomic DNA from *Hydra magnipapillata* was assayed for by enzymatic hydrolysis followed by labeling with either ^32^P or BODIPY. Both approaches respectively yielded m^5^dC frequencies of 2.3% and 2.6–3.1% indicating ∼1 in 40 cytosines are methylated in the *H. magnipapillata* genome (Hassel et al., 2010; Krais et al., 2010). Since the genome of *Hydra* has a low GC content, the level of non-CpG methylation might be significant and the location of the modifications in the gene bodies may play a role in transcriptional regulation as seen in some other animal species. The higher level of m^6^dA in the *Hydra* genome might correspond to the AT-richness of the *Hydra* genome (Chapman et al., 2010). Regions of DNA with higher AT content have been shown to harbor clusters of m^6^dA deposition in mammalian cells. In addition, the level of m^6^dA positively correlates with transcription (Pacini et al., 2019) and might facilitate the transcription required in the continuously dividing and differentiating cells of the *Hydra* body column cells. The sequence context and function of DNA methylation in *Hydra* are yet to be determined. Single-cell transcriptomics data (Siebert et al., 2019) may provide clues to the localization of m^5^dC and m^6^dA regulators. Orthologs of m^5^dC writers and erasers are highly upregulated in the female germline, suggesting that m^5^dC may be dynamically regulated for oocyte reprogramming, while a high level of its reader (MBD2) could function to silence oocyte chromatin (Figure 6A). In contrast, moderate DNMT3a levels observed in differentiating cells coupled with low TET2 levels may correspond to an increase in cytosine methylation during differentiation. DNMT1 appears to be high in stem cells as evident from the scRNA-seq analysis, concomitant with its role in methylation maintenance in mitotic cells (Bestor and Ingram, 1983). Putative transcripts of proteins involved in m^6^dA metabolism are similarly enriched in the female germline and low in terminally differentiated cells (Figure 6A). Mitotic accumulation of m^6^dA by DNA polymerase could potentially counteract the overall low methyltransferase levels toward the maintenance of the mark (Musheev et al., 2020). The observed high levels of the putative m^6^dA demethylase ALKBH1 (Wu et al., 2016), particularly in differentiated *Hydra* cells may ensue lower m^6^dA levels.

Contemporary studies using other cnidarians may provide some clues and motivation toward understanding the biological role of DNA methylation. There is a large level of variation in the level of methylation across non-bilaterian genomes (de Mendoza et al., 2019). A comprehensive study on a large cohort of eumetazoans reported an m^5^dC frequency of 1.8% in *N. vectensis*, predominantly in the C_*p*_G context. Additionally, 1 in 1000 cytosines were found methylated in the anemone’s mitochondrial DNA (Zemach et al., 2010). Reanalysis of the *Nematostella* cytosine methylome revealed a characteristic bimodal distribution of genes with low and high C_*p*_G abundances, though no significant correlation with expression level was observed (Nanty et al., 2011). C_*p*_G methylation occurs mutually exclusive of H3K4me3 in *Nematostella*, and hence the latter may inhibit the methylation of the underlying DNA as it does in bilaterians (Schwaiger et al., 2014). Studies on corals suggest gene body C_*p*_G methylation promotes gene expression rigidity (Dixon et al., 2014), codon selection (Dixon et al., 2016), transcriptional homeostasis (Li et al., 2018), and adaptation to high temperature (Dixon et al., 2018) or low pH (Liew et al., 2018). Interestingly, Myxosporea, another class of cnidarians, lacks both the DNMTs and cytosine methylation as identified by a study on the genomes, transcriptomes, and proteomes of the various cnidarian species (Kyger et al., 2021).

A study on another pre-bilaterian, the ctenophore *Pleurobrachia bachei* (Pacific sea gooseberry), determined an m^5^dC frequency of ∼1%. DNMT and TET orthologs were expressed during early developmental stages, with high TET expression also found in adult combs. This may be functionally correlated with the presence of its intermediate product–hm^5^dC–in the *P. bachei* genome (Moroz et al., 2014). Non-CpG DNA methylation is also seen in the genome of *Mnemiopsis*, another Ctenophore at a low level of 0.11% and the distribution is similar to that in honey bees (Dabe et al., 2015). A recent comparative study of genome-wide DNA methylation at the root of the animal phyla has thrown light on the conserved mechanisms of DNA methylation at the root of animal evolution. Here, authors have shown that molecular players involved in DNA methylation and demethylation such as DNMTs and TETs are conserved in non-bilaterian invertebrates except in Placozoa. This study has clearly demonstrated the occurrence of hypermethylated genomes, especially in *Amphimedon* (a marine sponge) similar to vertebrates indicating convergent evolution (de Mendoza et al., 2019). However, further detailed studies are required to elucidate the DNA methyl code both at CpG and non-CpG locations in the context of gene regulation and other functions.

## RNAi in *Hydra*

### miRNAs and Endo-siRNAs

**FIGURE 7:**
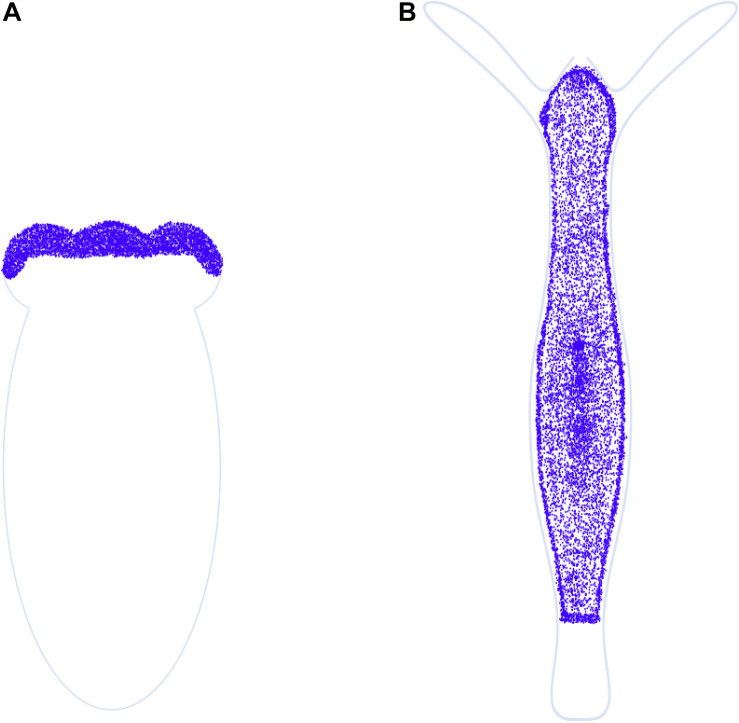
miR-2030 comparative expression profile. Schematic representation of miRNA *in situ* hybridization data for miR-2030 in **(A)** *N. vectensis* (primary polyp) and **(B)** *H. magnipapillata*, hybridized with DIG-labeled probes complementary to it (Adapted respectively from Moran et al. (2014) and Krishna et al. (2013); scale bars unavailable). Divergent localization of miR-2030 between the two cnidarians is observed, with the miRNA expressed at the oral end of *Nematostella* but excluded from the oral and aboral ends of *Hydra*. miR-2030 may have different targets in the two organisms, as predicted by an ortholog search for the *Nematostella*-specific miRNA target in *Hydra* yielding only ∼33% homology with 42% query coverage. This presumably explains the divergent localization.

The development of next-generation sequencing technologies facilitated transcriptomic profiling of tissue samples. An early platform called Roche 454^TM^ pyrosequencing was employed to analyze the small RNA (sRNA) complements of 13 metazoan species, including *H. magnipapillata*. Four miRNAs were found in the freshwater cnidarian, all novel, including the cnidarian-specific *miR-2022* in common with *N. vectensis* (Grimson et al., 2008; Wheeler et al., 2009). The *H. magnipapillata* genome sequencing project also identified ≥17 *Hydra* miRNAs-including the cnidarian-specific *miR-2022* (Chapman et al., 2010). The first comprehensive *Hydra* sRNA analysis, performed by deep sequencing using the Illumina^TM^ platform, identified 238 unique 21–22 nt long miRNAs mapping to 126 loci, only 3 of which (*miR-2022*, *miR-2029*, and *miR-2030*), were in common with *N. vectensis*. However, barely half the sRNA reads mapped to the draft *H. magnipapillata* genome, which may be attributed to inadequate coverage of the latter and could explain why only 17 miRNAs were previously identified (Krishna et al., 2013). Another study reported a similar genome-transcriptome disparity in *H. vulgaris* Basel, having employed both Illumina^®^ and Roche 454^®^ sequencing platforms (Wenger and Galliot, 2013). Comparative analysis of sRNAs expressed in both whole and regenerating *H. magnipapillata* polyps yielded differential expression of 10 miRNAs, three of which were validated by qPCR analysis. Additionally, analyses of *H. vulgaris* AEP and *H. vulgaris* Ind-Pune sRNAs revealed that unlike in bilaterians, miRNA conservation is low across *Hydra* species. ∼20% of the miRNAs identified exhibited perfect complementarity in their stem region, which is a characteristic of siRNAs and were termed endo-siRNAs. An enrichment of purines on the 5′ ends of *Hydra* miRNAs was observed, suggestive of cleavage by Dicer (Krishna et al., 2013). Analysis of the miRNA dataset generated by Krishna et al. (2013) determined that *Hydra* miRNAs frequently undergo 3′ U or A-tailing after the 23rd base, and additionally identified conserved terminal uridyltransferases that may catalyze the same. This was also found to be the case for *N. vectensis* (Modepalli and Moran, 2017). Additionally, homologs of key miRNA pathway components have been identified in the transcriptomes of *Hydra* and the anthozoans *Nematostella* and *Acropora*. The miRNA components in Cnidarians exhibit very specific expression patterns and putative roles during the development of the organism. The mechanism of action of these molecules appears to be more similar to that in plants than in bilaterian animals based on their target complementarity, mode of gene regulation, and post-transcriptional modifications (Moran et al., 2014). Notably, cnidarians express HYL1, which is a partner of Dicer typically employed by plants (Grimson et al., 2008; Krishna et al., 2013; Moran et al., 2013). Another striking similarity between plant and cnidarian miRNAs is their near-perfect complementarity to target transcripts, which promotes transcript cleavage instead of ribosome stalling. Concomitantly, cleavage products for 2 out of 5 putative *H. magnipapillata* miRNA targets consistent with miRNA action have been detected. Nematogalectin-related 2 mRNA was determined to be a target of cnidarian *miR-2022*, and both were observed to co-localize in the nematocytes of *N. vectensis* (Voinnet, 2009; Moran et al., 2014). Interestingly, the localization of *miR-2030* in primary polyps of *N. vectensis* was found to differ dramatically from that observed in *H. magnipapillata* (Figure 7). The closest homolog of the uncharacterized *N. vectensis* protein encoded by the target gene of *miR-2030* (LOC5501396)–found by BLAST in *H. magnipapillata*–shared only ∼33% sequence identity with a query cover of 42%. It is thus possible that *miR-2030* regulates a different protein in *Hydra*, which could explain the divergence in its localization between the two cnidarians if found to be the case. miRNA pathway components (including HYL1) have also been detected in corals, with predicted roles in endosymbiosis, biomineralization, thermal, and pH stress responses (Liew et al., 2014; Gajigan and Conaco, 2017; Baumgarten et al., 2018; Urbarova et al., 2018). Taken together, these studies are suggestive of a common ancestral miRNA mechanism that diverged in higher metazoans, presumably enabling more rapid and dynamic post-transcriptional responses to stimuli (Gu et al., 2018).

Knockdown of genes in *Hydra* by RNAi is a common experimental practice, suggesting that the cnidarian harbors the functional machinery to effect the same. However, functional studies on this gene regulation system as well as miRNA functions are currently lacking. A study in *N. vectensis* has shown that zygotic perturbation of the sRNA 3′ methyltransferase Hua Enhancer 1 (HEN1) negatively impacts the stability of its miRNAs and piRNAs, and phenocopies the developmental arrest (at the larval stage) observed on RNAi of Dicer1 and PIWI2 (Modepalli et al., 2018). The Ago2 component of the RNAi machinery shows a significantly higher expression in the endodermal lineage relative to the ectodermal lineage (Figure 6C). Similar perturbation studies in *Hydra* could be useful for understanding the role of miRNAs in its unique biology.

### piRNAs

Sequencing of the *H. magnipapillata* genome uncovered a few piRNA-like sRNAs (Chapman et al., 2010). Homologs of piRNA pathway components PIWI and Vasa have been detected in the germline and interstitial stem cells in *Hydra* (Mochizuki et al., 2001; Hemmrich et al., 2012; Nishimiya-Fujisawa and Kobayashi, 2012). The sRNA-seq analysis by Krishna et al. (2013) was the first to identify 27–29 nt long piRNA-like RNAs as being the most abundant sRNA species in *H. magnipapillata*, mapping to transposable elements, expressed sequence tags, and non-coding regions. The majority of the putative piRNAs mapping to transposons exhibited the characteristic ping-pong signature of a 5′ uracil with a 10th position adenosine, while the minority was only enriched for uracil at their 5′ ends-suggestive of Ago-mediated cleavage. piRNA-like RNAs were also found mapping to mRNAs. Notably, potential piRNAs that mapped onto histone genes reduced in abundance during head regeneration while the histone-encoding transcripts were themselves upregulated at the corresponding time points, suggesting that these sRNAs may regulate histone abundances. Multiple other piRNA-like RNAs are also differentially regulated during head regeneration with putative roles in regulating the expression of target genes in the regenerating tissue (Krishna et al., 2013). Given that the piRNA-PIWI protein complexes contribute to transcription gene silencing, it is plausible that piRNA-mediated gene regulation may affect the transcription of histones and other genes. The observed target gene upregulation corresponding to piRNA-like RNA downregulation implies that such transcriptional silencing is reversible, at least in regenerating *Hydra* tissue. Additionally, differentiated *Hydra* cells may employ other gene regulatory mechanisms including histone methylation to repress genes.

The putative piRNA pathway in *Hydra* has been characterized by independent studies. piRNA pathway components are enriched within characteristic perinuclear foci in interstitial and female germline stem cells. In mature male germline cells, however, only a single punctum was visible-resembling chromatoid bodies observed and characterized in higher metazoans as sites of stable transposon repression by DNA methylation. *Hydra* PIWI homologs Hywi and Hyli, respectively interact with primary and secondary piRNAs, but not miRNAs, which may enable their ping-pong amplification toward transposon silencing. Additionally, both these homologs possess critical symmetrical dimethylarginine residues at their N-termini (Lim et al., 2014). Somatic functions have also been demonstrated for Hywi, with lineage-specific non-transposon RNA targets also identified. *Hywi* RNAi lines disintegrate within 12 days of hatching due to epithelium disruption and are impaired in transposon silencing, confirming the functionality of piRNAs in transposon repression in somatic cells (Juliano et al., 2014; Teefy et al., 2020). The scRNA-seq data also corroborates the presence of the various piRNA interacting machinery in both germline stem cells, somatic stem cells, and few somatic differentiated cells as well (Figure 6C). The somatic presence of PIWI was previously observed in another hydrozoan, *Podocoryne carnae* (Seipel et al., 2003). The pathway may promote interstitial cell differentiation in *H. echinata*, as evidenced by compromised head regeneration on knockdown of *piwi* or *vasa* (Bradshaw et al., 2015). piRNA pathway components have also been detected in *N. vectensis* and were similarly found to be enriched in perinuclear granules in both germline and somatic cells. piRNAs are the predominant sRNA population in the anemone, mapping to transposons and also to protein-coding genes, the latter set being mutually exclusive of genes targeted in *Hydra* (Grimson et al., 2008; Praher et al., 2017). piRNAs are also abundant in other anthozoans such as *Stylophora pistillata* and *Anemonia viridis* (Liew et al., 2014; Urbarova et al., 2018). Taken together, these studies outline roles for the cytoplasmic piRNA pathway in both germline and somatic cnidarian stem cell lineages, which may respectively restrict mutagenesis during gametogenesis and clonal propagation and, to some extent, regulate gene expression. The somatic function of PIWI is therefore ancient and may have been lost in few higher metazoans, while nuclear functions of the germline piRNA pathway appear to have evolved later.

## *Cis*-Regulatory Elements

Multicellular organisms are an ensemble of different cell types with distinct functions orchestrated spatially to achieve a phenotype. The development of these organisms from a single cell requires precise regulation of cell fates in a spatiotemporal manner. *Cis*-regulatory elements (CREs) facilitate coordinated regulation of various sets of genes required for development and physiology (Levine, 2010). These elements are short DNA sequences (mostly, non-coding) that are bound by TFs or other regulatory molecules and influence gene regulation (Ong and Corces, 2011). Typically, CREs can be categorized into promoters and enhancers. Promoters are regions with overlapping TSSs and contain single to multiple CREs that allow binding of the transcription initiation complex to attain gene transcription (Lenhard et al., 2012). Enhancers are located at varying distances from their target genes and amplify the transcription by regulating the promoter (Shlyueva et al., 2014). Enhancers play a crucial role in context-specific gene regulation and mutations in these regions lead to altered morphology or physiology. Due to this reason changes in enhancer sequences often result in the evolution of novel functions (Carroll, 2008; Frankel et al., 2011). Therefore, it is important to understand the evolution of such CREs associated with eumetazoan-specific functions. However, there is very little information available regarding their nature and regulation at the base of Eumetazoa where major transitions such as cell type and phenotype evolution occurred.

**FIGURE 8:**
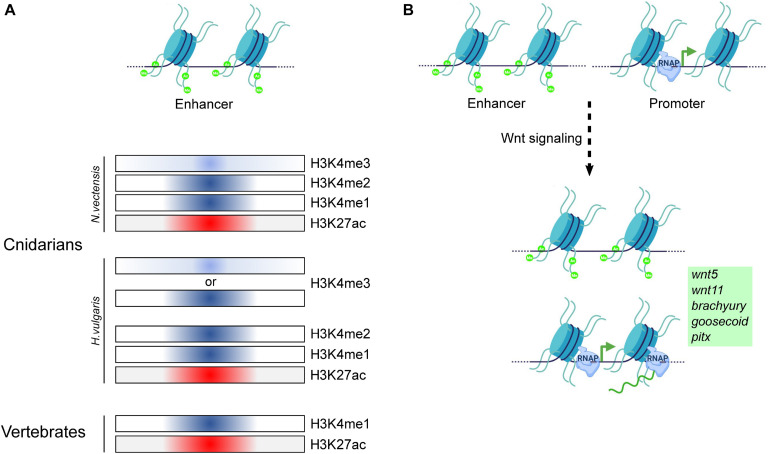
Regulation of gene expression by *cis*-regulatory elements in Cnidaria and Vertebrata. **(A)** Comparison of the occupancy of various histone modifications on putative enhancer regions in Cnidarians and vertebrates. The classical marks associated with enhancer regions in vertebrates have been identified to localize to putative enhancer regions in both *N. vectensis* and *H. vulgaris*, indicating conservation of histone mark function in both the groups of the animal kingdom for gene regulation. **(B)** Putative enhancer regions have been identified to be regulated by the activation of the Wnt/β-catenin signaling pathway, resulting in target gene expression in *H. vulgaris*. Model based on data from Schwaiger et al. (2014) and Reddy et al. (2020a).

A first step toward understanding the evolution of enhancers is the identification of CREs in genomes of early diverging eumetazoan phyla such as Cnidaria. Characteristic features such as open chromatin and histone marks including H3K27ac, H3K4me1, and H3K4me3 facilitate the identification of CREs. Their functional relevance can be elucidated by studying their dynamics under different experimental conditions. Toward this, a study was performed in *N. vectensis* in which ∼5000 enhancer elements were identified. Here, a combination of p300 binding regions, H3K27ac, H3K4me1, H3Kme2, and H3K4me3 were used to predict the enhancers. This study indicated that bilaterian enhancer characters are conserved in *N. vectensis* (Schwaiger et al., 2014). Among these, ∼600 enhancers have been shown to play a role in transcription regulation associated with the circadian rhythms (Weizman et al., 2019). In *Hydra*, Wnt signaling has been shown to play a vital role in the organizer formation and is associated with massive transcriptional changes (Reddy et al., 2019). This phenomenon is crucial in the evolution of body axis patterning. In a recent study by Reddy et al., the genome-wide dynamics of enhancer elements were studied based on the occupancy profiles of activation-specific histone marks. This study has revealed that upon activation of Wnt signaling at least ∼11000 intergenic CREs exhibit differential H3K27ac modification which is associated with the enhancer activity (Figure 8B). Further, the comparative analysis predicted that ∼700 genes are regulated by these enhancers. The list of these genes includes many of the important transcription factors such as *CnGsc*, *HyBra*, *CnAsh*, *CnOtx* that play a seminal role in the head organizer formation. This study provided significant insights into the enhancer-mediated regulation of organizer genes (Reddy et al., 2020a). *Hydra* is known for its extraordinary regeneration capacity and organizer formation is a critical step in the head regeneration process. A recent study used assay for transposase-accessible chromatin (ATAC)-seq and histone marks to identify the enhancers involved in the regeneration process. This study identified ∼3000 enhancer elements that might contribute to gene regulation during regeneration. Murad et al. (2019) further demonstrated modulation of chromatin opening in the *HyWnt3a* gene locus occurring as early as 4 h post-amputation. Notably, enrichment of *CnGsc* binding motifs was observed on the enhancer elements found during the regeneration time course which is important in head patterning in *Hydra*. Based on the initial studies, H3K27ac has proven to be a reliable histone modification mark to identify the enhancer elements with more confidence. However, in *Hydra*, H3K4me3 also exhibits colocalization at the enhancer elements which needs to be further studied. Additional efforts are required toward the identification of the enhancer elements involved in precise functions such as cell type determination and their functional validation. The conservation of the occupancy of histone marks is depicted in Figure 8A.

## Chromatin Organization

Studies in the 1960s and 1970s first reported the morphology of cells in various *Hydra* spp. (Lentz, 1965; Weissman et al., 1969; Moore and Dixon, 1972; Mookerjee, 1974). Interstitial cells in *Hydra littoralis* were observed to have electron-dense chromatin at the nuclear periphery consistent with heterochromatin, and exhibited chromatin aggregation and increased nucleolar size during somatic differentiation (Lentz, 1965). In spermatids of *H. littoralis*, a close association of the peripheral chromatin with basally located mitochondria and the apical plasma membrane during chromatin condensation results in an hourglass-shaped heterochromatin profile, which may be attributed to the requirement of ATP and Ca^2+^/Mg^2+^ by chromatin condensation machinery (Weissman et al., 1969). Surprisingly, while embryonic depletion of the Lamin protein was found to be lethal in *H. vulgaris* AEP, its inducible depletion and overexpression did not affect growth and interstitial cell proliferation, despite causing lamina defects. This robustness to lamina perturbation is attributed to fewer lamina-binding proteins compared to higher metazoans (Klimovich et al., 2018). Vertebrate embryonic stem cells are also indifferent to Lamin depletion, highlighting their dispensability in stem cells across metazoans (Stewart and Burke, 1987; Kim et al., 2011). Chromosome territories and evolutionarily conserved patterns and sizes of DNA replication foci have also been identified in *H. vulgaris* stem cells (Alexandrova et al., 2003).

Assay for transposase-accessible chromatin-seq mapping of the *H. vulgaris* genome revealed >27,000 “open” exons, introns, promoter-proximal and intergenic regions across homeostatic, budding, and regenerating polyps. Among these, the promoter-proximal regions exhibited the highest accessibility, followed by the intergenic ones. The accessibility of the *wnt3a* locus that encodes the head organizer morphogen Wnt3a in the regenerating tissue, was found to peak 4 h post-decapitation at its promoter and downstream enhancer regions. A similar accessibility pattern for *wnt3a* was observed in the hypostome, in agreement with its role in head organizer maintenance (Murad et al., 2019). Open chromatin profiles have been detected at the promoters of 2000 highly expressed genes in *H. vulgaris* AEP polyps. A strong accessibility peak ∼5 Kb upstream of the *wnt3a* TSS is observed in whole polyp chromatin compared to hypostome and regenerating tissue chromatin (Siebert et al., 2019). This may be due to the under-representation of the hypostome/regenerating tip chromatin fraction in the whole polyp chromatin, suggesting that the openness of this region may reduce significantly in the hypostome. It is plausible that this upstream region is a silencer of *wnt3a* and may be bound by head repressors such as Sp5 in non-hypostome cells (Vogg et al., 2019). This hypothesis may be tested by performing ChIP of Sp5 followed by quantitative PCR for the upstream region in whole polyps versus hypostome tissue, while a functional validation mandates deletion of the putative silencer. ATAC-seq mapping in neurons of the cnidarian *N. vectensis* revealed neuron-specific gene accessibility patterns that correlated with neuronal transcription profiles (Sebé-Pedrós et al., 2018). A positive correlation between gene accessibility and transcription has been observed for most circadian rhythm-regulated gene promoters in the starlet sea anemone *N. vectensis*, which also has distal regions with enhancer-like properties, and thermal stress-responsive genes in the coral *Aiptasia pallida* (Weizman and Levy, 2019; Weizman et al., 2019). In a recent study, Reddy et al. (2020b) showed that unlike in higher metazoans, RNA Pol II does not pause at the TSSs of most genes in *H. vulgaris* Ind-Pune and is instead uniformly distributed across gene bodies, with its occupancy positively correlated with the expression level. Moreover, the <250 genes that show Pol II pausing exhibit strongly positioned +1 nucleosomes, superior expression, and, primarily, an involvement in gene expression and translation. Unlike earlier divergent phyla, all negative elongation factor complex (NELF) homologs were identified in *H. vulgaris*, suggestive of the mechanistic origin of RNA Pol II pause in cnidarians (Reddy et al., 2020b).

**FIGURE 9:**
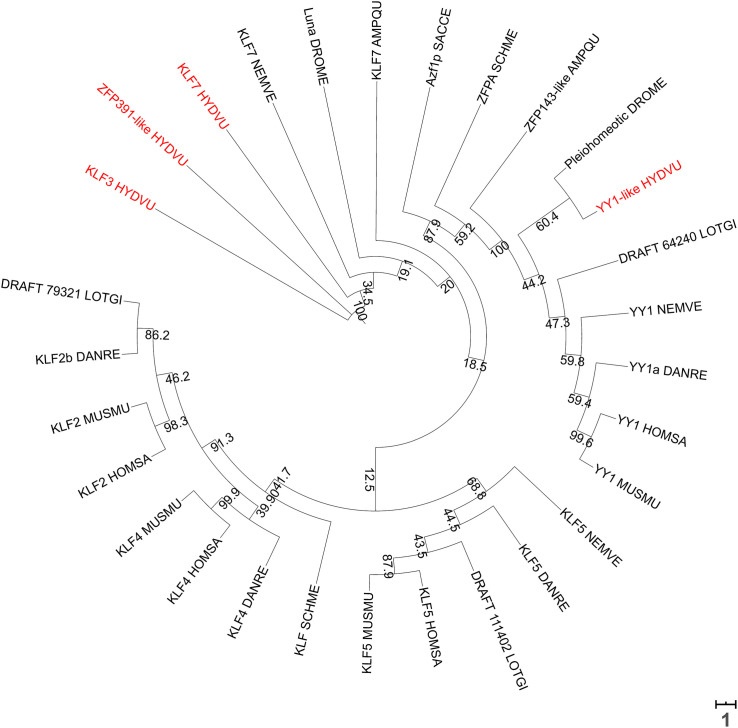
A phylogenetic tree of KLF2/4/5 and YY1 orthologs from various species prepared using NGPhylogeny.fr (Lemoine et al., 2019), with 0.1×-scaled bootstrap values displayed at each node. Branch lengths are proportional to the tree scale bar. Sequences were aligned with MUSCLE, followed by trimAI curation and then PhyML phylogeny with 1000 iterations. The tree was visualized in a circular format using iTOL (Letunic and Bork, 2019). Scale bar represents the number of substitutions per site. *Hydra* KLF3 and KLF7 cluster together and in the same clade as Nematostella KLF7, while *Hydra* YY1 clusters with the other YY1 orthologs. Names of all *Hydra* proteins are indicated in red.

The presence of chromatin loops and higher-order assemblies such as the topologically associating domains (TADs) is yet to be determined in polyps. RNA Pol II was detected at putative enhancers in *N. vectensis*, suggestive of enhancer-promoter looping (Schwaiger et al., 2014). Cnidarians typically do not encode orthologs of the chromatin organizers CTCF (Heger et al., 2012; Zimmermann et al., 2020) and SATB1/2 (current analysis) family proteins. There is evidence to suggest that CTCF-less genomes do not form TADs, which are recurring chromatin domains first evidenced by Hi-C data (Dixon et al., 2012; Kenny et al., 2020). The role of CTCF in invertebrate animals is also not well established since it is dispensable for the maintenance of TAD architecture (Kaushal et al., 2021). TADs may therefore be an architectural feature unique to bilaterian genomes although this phenomenon may be independent of known homologs of CTCF. On the other hand, hydrae do possess YY1 and KLF2/4/5 orthologs. *Hydra* YY1 might participate in an evolutionarily conserved interaction with PRC2 proteins and bind to CCAT-motifs within Polycomb DNA motifs. HyYY1 was observed to be enriched in the nuclei of ectodermal epithelial cells in the gastric column, head and foot regions, suggestive of a region-independent role. However, it was also detected on the membranes of these cells, though this is likely an artifact of antibody binding to non-YY1 membrane proteins. Unlike the YY1 protein, its encoding transcript was localized in interstitial cells, and PRC2 members EZH2 and EED showed a similar mRNA-to-protein localization disparity. However, the histone marks installed by PRC2 (H3K27me3) are detected only in the interstitial cells, and therefore the detection of Polycomb proteins in ectodermal epithelial cells is counterintuitive (Matt, 2011). KLF3, KLF7, and KLF8 are orthologs of human KLF2/4/5 that were detected as signatures of specific stem cell types in *Hydra*. KLF7 is enriched in epithelial stem cells, KLF8 is specific to ectodermal epithelial stem cells and KLF3 is found in both interstitial and ectodermal epithelial stem cells (Hemmrich et al., 2012). Phylogenetic analysis revealed that these *Hydra* KLF orthologs cluster with their respective non-vertebrate orthologs (Figure 9). However, the high sequence divergence between KLFs provides little ortholog predictive power (data are not shown), and therefore zinc finger proteins other than KLF3/7/8 could equally function as chromatin organizers. In contrast, YY1-like clusters with other YY1 orthologs, supporting its role as a putative chromatin organizer. Further, while orthologs the ATP-dependent chromatin remodelers Brahma (BRM), Sucrose Non-Fermentable 5 (SNF5), Chromodomain Helicase DNA-binding protein 3 (CHD3), and INO80 are predicted in *Hydra* (BLAST^®^ searches), they have not been characterized so far.

**FIGURE 10:**
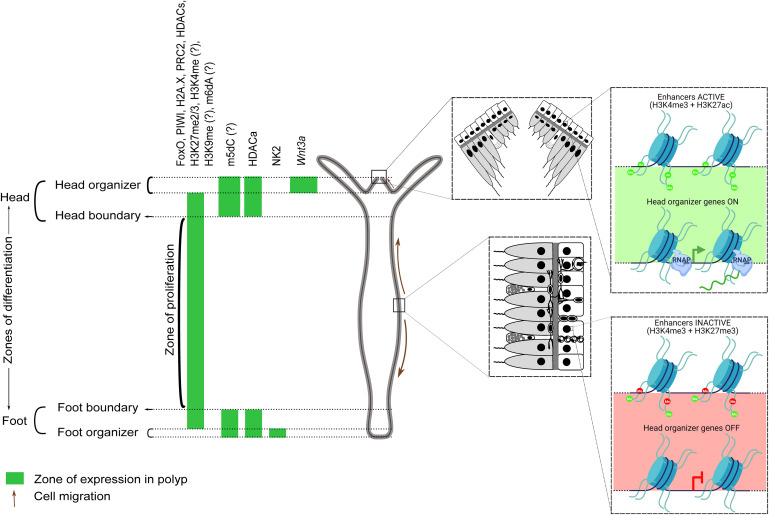
A graphical summary of the verified and scRNA-seq predicted roles of epigenetic regulators and histone modifications in Hydra. The head, gastric column, and foot regions are respectively demarcated by the expression of the master regulators Wnt3a (and other proteins), FoxO, and NK2. Epigenetic regulators enriched in the gastric column promote stem cell maintenance, while those at the apicobasal termini promote differentiation. HDAC activation (HDACa) has been shown to maintain the boundaries between these three regions. Regulators/modifications with a question mark are predicted based on the scRNA-seq data. Enhancers controlling head organizer genes are active in the head and repressed elsewhere by H3K27me3. Demethylation and acetylation of H3K27 switches on head-specific enhancers and the activated HDACs could be acting on the genes that play a role in proliferation and body-column maintenance. Head organizer genes become active at the hypostome, which may reflect head enhancer activation in ectodermal cells (inset). H3K27 modification-dependent enhancer regulation is also relevant in the context of Hydra regeneration, wherein the activation of head organizer genes post decapitation facilitates head formation at the regenerating tip.

Most of the studies on epigenetic modifiers focus on monitoring the transcript levels of the proteins with an evident disparity in mRNA-protein localizations for a few of them. Studies that investigate protein localization and interactions directly will provide better insight into the role of the resultant modification and regulatory nucleic acids. Additionally, single-cell ATAC-seq could provide the chromatin accessibility profiles of individual cells (Buenrostro et al., 2015), which could then be compared with the corresponding gene expression profiles from the *Hydra* scRNA-seq data toward identifying cell type-specific regulatory elements. Wherever information in addition to the scRNAseq is available for many proteins and modifications, we have speculated their role in the unique physiology of *Hydra* and its regenerative capacity. Further validations involving protein levels, the activity of the proteins, the dynamics of target modifications, and the post-transcriptional mechanisms are necessary to understand the complex epigenetic regulation of the developmental processes and regeneration in *Hydra*. The landmark studies in *Hydra* alluding to the above are listed in Table 1. A graphical summary of the validated and predicted functions of epigenetic regulators in *Hydra* is provided in Figure 10.

**TABLE 1:** Tabular summary of the epigenetic regulation studies in *Hydra* are reviewed here.

Epigenetic regulator	Presence	Function	References
Histone variants	Confirmed	H2A.X may protect genome integrity in *Hydra* stem cells H2B.6 has a putative role in chromatin compaction during spermatogenesis.	Reddy et al., 2017
Histone methylation	Confirmed	H3K4me2/3 marks active gene regulators; H3K27me2/3 may promote interstitial cell differentiation and silence enhancers	Genikhovich et al., 2006; Khalturin et al., 2007; Matt, 2011; Murad et al., 2019; Reddy et al., 2020a
Histone acetylation	Confirmed	H3ac promotes stem cell maintenance; H3K27ac marks active gene regulators	Murad et al., 2019; López-Quintero et al., 2020; Reddy et al., 2020a
Chromatin remodelers	Orthologs detected	N.D.	BLAST^®^
Chromatin organizers	Confirmed	YY1 may recruit PRC2 to gene regulatory elements; KLF3/7/8 (to be characterized) enriched in specific stem cell lineages	Matt, 2011; Hemmrich et al., 2012
Cytosine methylation	Confirmed	N.D.	Hassel et al., 2010; Krais et al., 2010
Adenosine methylation	Confirmed	N.D.	Krais et al., 2010
Endo-si/miRNAs	Confirmed	N.D.	Wheeler et al., 2009; Chapman et al., 2010; Krishna et al., 2013; Moran et al., 2013
piRNAs	Confirmed	Repress germline and somatic transposons; May regulate histone levels	Chapman et al., 2010; Hemmrich et al., 2012; Nishimiya-Fujisawa and Kobayashi, 2012; Krishna et al., 2013; Juliano et al., 2014; Lim et al., 2014; Teefy et al., 2020

*Abbreviations: BLAST, Basic Local Alignment Search Tool; N.D., not determined.*

## Future Perspectives

Apart from the studies suggested in each section of this review, the following investigations may further our understanding of the epigenetic regulation and its transgenerational inheritance in *Hydra*:

1. Mapping the epigenomes of *Hydra* sperm and oocyte to identify the conserved determinants of transgenerational inheritance. Here, external fertilization is advantageous toward the collection of gametes.
2. Delineating the epigenetic regulation of cell fate determination and axis patterning in homeostatic and regenerating polyps could provide evolutionary insights into the process.
3. Performing high-resolution proteomic analyses at a single cell level (Ma et al., 2013) will facilitate further understanding of the dynamics of the epigenetic regulators since many of them are enzymes and their protein levels are more relevant to the regulation of cellular and physiological functions.
4. The dynamics and effects of DNA methylation have not been investigated in detail in *Hydra*. The continuous proliferation and differentiation of cells in *Hydra* offers an excellent paradigm to gain insights into the ancestral roles of the DNA modifying machinery during metazoan evolution. DNA methylation changes are associated with aging and age-related diseases. *Hydra* is an immortal organism and it does not exhibit typical aging-related disease phenotypes. Thus, understanding the maintenance of DNA methylation in *Hydra* will provide insights into the aging process.
5. Recent studies have elucidated the conservation of *cis*-regulatory DNA elements in the regulation of developmental genes across multiple canonical model organisms (Plessy et al., 2005; Wong et al., 2020). Investigating the conserved nature of *Hydra* enhancer elements in higher organisms will allow us to understand the fundamental roles of these regulatory regions and how they were adapted into distinct signaling pathways as the processes diverged. The redundancy of enhancer-mediated regulation can also be addressed well in organisms such as *Hydra*. They exhibit the processes of embryonic development, budding, and regeneration which offer an opportunity to look at the regulation of the same genes in diverse physiological contexts. Especially, close phylogenetic relationship with Bilateria and occurrence of conserved molecular machinery, cell types, and developmental processes provides a unique opportunity to identify the conserved *cis*-regulatory elements responsible for the evolution of eumetazoan cell types and functions.
6. The organization of chromatin into active and inactive compartments aids in the efficient regulation of gene expression in a spatiotemporally appropriate manner. Early metazoans including *Hydra* lack the CTCF-based higher-order compartmentalization mechanisms. However, other mechanisms based on the PcG components, transcription-based organization, and arrangement of the genomic regions in an alternating active-inactive format contribute toward achieving this (Matthews and White, 2019). It is of immediate interest to dissect the molecular mechanism/s of determining the topological domains and the higher-order 3D architecture of the *Hydra* genome.
7. Along with the chromatin level regulation of gene expression, there is an additional level of post-transcriptional checkpoints in the cells. A part of this process has been discussed in the review and the other part involves chemical modification of the RNA species inside the cells. Various types of RNA modifications are reported, and the occurrence of the *ADAR* machinery has been identified in organisms at the root of animal phyla such as Ctenophores, Placozoans, sponges, and Cnidarians (Keegan et al., 2017). Among the RNA modifications, an important modification is the m^6^A on different RNA species in the cell which targets it for degradation or stabilizes it for translation (Zaccara et al., 2019). The various components of the modification machinery including the writers, erasers, and readers of this modification in *Hydra* have not yet been identified and the role of these molecules and the modified RNA in the physiology of *Hydra* will be interesting to study in the context of cellular differentiation during regeneration and budding.

## Author Contributions

AP and AG contributed to design concept, collected and analyzed literature, wrote bulk of the manuscript, and prepared table and figures. PCR and SG provided critical inputs as the corresponding authors, wrote sections of the manuscript, and obtained funds. All authors approved the submitted version.

## Conflict of Interest

The authors declare that the research was conducted in the absence of any commercial or financial relationships that could be construed as a potential conflict of interest.

## Abbreviations

ALKBH: AlkB Homolog
ATAC/ChIP/scRNA-seq: assay for transposase-accessible chromatin/chromatin immunoprecipitation/single-cell RNA-sequencing
bp: base pair
BODIPY: boron-dipyrromethane
C_*p*_G: cytosine-phosphate-guanosine
m^6^dA: *N*^6^-methyldeoxyadenosine
m/hm^5^d: 5-methyl/5-hydroxymethyldeoxycytosine
nt: nucleotide
PIWI: P-element Induced Wimpy testis
Pol: polymerase
RLM-RACE: RNA ligase-mediated rapid amplification of cDNA ends
RNAi: RNA interference
s/mi(R)/endo-si/piRNA: small/micro/endogenous-short interfering/PIWI-interacting RNA
TAD: topologically associating domain
TSS: transcription start site
UTR: untranslated region.
